# Personal

**Published:** 1892-11

**Authors:** 


### Personal.

Dr. John Parmenter, late of 372 Franklin street, has removed his office and residence to 116 North Pearl street, Buffalo. His office hours are from 8 to 10 A. M., and from 2 to 3 P. M.

Dr. J. Collins Warren, of the Harvard Medical School, has accepted the Executive Presidency of the Section of Medical Pedagogics in the Pan-American Medical Congress, vice Dr. D. B. St. John Roosa, declined. This is an admirable appointment, and Dr. Warren will not fail to make this section what it should be, namely, the most important department of the Congress.

Dr. Charles E. Sajous, editor of the *Annual* of the *Universal Medical Sciences*, has been appointed a Knight of the Legion of Honor of France, for his services to the French colony and to science, as an American of French descent. Dr. Sajous has taken up his residence at No. 28 rue de Madrid, Paris, whence he will continue the conduct of the *Annual*, and will also translate it into French.

